# The Treatment of Cough with Heroin

**Published:** 1902-02

**Authors:** 


### ABSTRACT.

### The Treatment of Cough with Heroin.

HENRY HERMAN, New York, (*Denver Medical Times*, May, 1901,) says that heroin has now been tested in such a large number of cases, by such trustworthy observers that its status is becoming fixed with certainty. He thinks the result of clinical and pharmacological experience is to designate heroin as a respiratory sedative superior in every respect to the older narcotics, and, withal, devoid of toxic or depressant properties. Its physiological action consists in a diminution of the frequency of respiratory movements, and at the same time in an increase of the volume of the individual respiratory act. The reduction of the tussal reflexes is combined with the relief of pain without the danger of a drug habit, which exists when other opiates are prescribed.

In regard to the after-effects he accepts the conclusions of Manges, that heroin is not followed by serious effects of an untoward character unless it be administered in excessive doses. His own observations, drawn from a large number of cases, tend to confirm this view.

There is, he thinks, a tendency to constipation, especially in old persons; easily combated, however, with mild laxatives. But it is to be remembered that heroin is a derivative of morphine, and therefore it is to be used with that caution and judgment, which drugs of this class demand. Fortunately its very efficiency precludes the necessity of administering doses of any magnitude beyond the usual.

Herman, in the course of his paper, reports a group of 19 cases of all kinds treated with glyco-heroin, which he classifies and comments upon as follows:

Of the 19 cases reported, there were 10 cases of whooping cough in patients ranging from 2½ to 38 years of age. In these the dose varied, according to age, from 10 drops to a teaspoonful, the intervals not exceeding four hours. The results were very satisfactory in all cases, with the single exception of case 4, a mild type of whooping cough in a patient aged 38 years, in whom nothing but a change of climate had the desired effect. In the other a case of pertussis, there was very prompt diminution of the number and severity of the paroxysms and an improvement in the general well-being of the patient. These effects were all the more noteworthy as they are rarely seen in pertussis on the administration of any other sedative remedy.

In the 3 cases of subacute bronchitis, the results were so prompt as to astonish the patients themselves. With due care the continuation of the catarrhal process into a chronic state was avoided.

In the two cases of cough, occurring during pregnancy, glyco-heroin proved very efficient. Of all forms of cough, sedatives are most urgently indicated in the irritable condition of the whole system which obtains in the pregnant state. In glyco-heroin I found a palatable combination which at the same time is free from the depressant properties of the other opiates.

There were four cases of chronic bronchitis, the age of the patients ranging between 27 and 81 years. As has been noted by other writers, heroin is not so well borne by the aged as it is by the young. The dose must therefore be kept down in old people, and it is for this reason that in one of the cases, No. 17, in which the patient was 81 years old, no results could be obtained. In contrast to this case stand the other two instances of chronic bronchitis in the aged (persons aged 73 and 78 years respectively,) in which the effect was all that could have been wished.

He says, in conclusion, that while there probably never will be a remedy which will act with equal efficiency in all cases, the results which I obtained with glyco-heroin in a number of difficult and obstinate cases of cough, enable me to express myself as fully satisfied that it is an efficient respiratory sedative and expectorant.

